# Identification and intra-genus conservation analysis of non-conventional peptides in hybrid poplar 84K

**DOI:** 10.48130/forres-0026-0004

**Published:** 2026-02-28

**Authors:** Ying-Da Chen, Jian Tang, Xin-Yue Wang, Xin Lin, Hao-Ran Liao, Hui Liu, Li-Na Mei, Liang-Jiao Xue, Ying Guo

**Affiliations:** State Key Laboratory of Tree Genetics and Breeding, Co-Innovation Center for Sustainable Forestry in Southern China, Key Laboratory of Forest Genetics & Biotechnology of Ministry of Education, Jiangsu Key Laboratory for Poplar Germplasm Enhancement and Variety Improvement, Nanjing Forestry University, Nanjing 210037, China

**Keywords:** *Populus*, Non-conventional peptides, Tissue-specific, Allele, sORFs

## Abstract

The functional roles of non-conventional peptides (NCPs) encoded by short open reading frames (sORFs) are increasingly recognized. However, their evolutionary conservation among closely related species remains largely unexplored. This study presented a genome-wide identification of NCPs in the hybrid poplar 84K (*P. alba* × *P. glandulosa*), and analyzed NCPs' sequence conservation across six sections of the *Populus* genus. Using LC-MS/MS with a custom six-frame-translated genome database, 516 conventional peptides (CPs), and 337 NCPs were indentified. NCPs exhibited distinct properties, including shorter length and lower molecular weight, compared to CPs. Tissue-specific expression patterns were prominent, with peptides functionally linked to photosynthesis in leaves, cell wall biosynthesis in stems, and nutrient uptake in roots. Allelic analysis revealed a parent-of-origin expression bias for over 10% of peptides, each set enriched in distinct metabolic pathways. Notably, NCP sequences were significantly less conserved than CPs across the genus, though specific conserved motifs were identified. This work provides the first systematic NCP resource for a model hybrid tree, establishing a foundational platform for leveraging peptide biology in molecular forestry and hybrid breeding.

## Introduction

As foundational structural and functional elements of terrestrial ecosystems, trees hold globally significant ecological and economic importance^[1]^. The carbon sequestration potential of forest trees within frameworks aligned with the Kyoto Protocol is currently being quantified, with preliminary assessments indicating substantial contributions. Concurrently, fast-growing tree species under sustainable management regimes (e.g., *Populus* spp.) play a pivotal role in meeting global demands for industrial timber, pulp for paper manufacturing, and other lignocellulosic biomass-derived products. *Populus* L., a typical representative of the Salicaceae family, can be divided into six major sections, including *Aigeiros*, *Leucoides*, *Populus*, *Tacamahaca*, *Turanga*, and *Abaso*, based on relative morphological similarity and crossability^[2]^. The genus *Populus* is full of contrasts and surprises. Following the successful sequencing of the *Populus trichocarpa* genome in 2006, poplar has become one of the most interesting and extensively studied model organisms^[3]^.

The sequencing and annotation of multiple poplar species and their hybrids have greatly facilitated genome-wide functional analyses in *Populus*^[4,5]^. For example, a pangenome study of 19 species revealed extensive structural variations and functional gene divergence, providing a robust framework for in-depth research on the functional genome of poplar trees^[6]^. The poplar genome typically spans ~500 Mb across 19 chromosomes, encoding 20,000−50,000 genes with > 95% sequence conservation among species^[3,7]^. Following the central dogma, genetic information is transcribed from DNA to mRNA and translated into functional proteins that regulate key biological processes in poplar trees, including wood formation, signal transduction, and the control of dormancy and flowering. Additionally, advanced research has revealed extensive translation of unannotated ORFs and non-canonical sequences in noncoding RNAs, expanding beyond current poplar genome annotations^[8]^. Research in model plants such as *Arabidopsis* and maize^[9]^ has shown that the translation of non-canonical genomic sequences is widespread, generating functional peptides that regulate growth, development and stress responses.

Conventional peptides (CPs), typically comprising 5−75 amino acids are translated from annotated gene regions. CPs from *Populus* such as PtrCLE10 can participate in regulating root development^[10]^; PtrCLE20, which was produced by developing xylem cells plays a role in regulating lateral growth by inhibiting the cambium activity of trees^[11]^; *PttCLE47* encoded peptide can promote the development of cambium and secondary xylem formation in hybrid poplar trees^[12]^. Unlike CPs, non-conventional peptides (NCPs) derive from diverse genomic elements, such as intergenic and intronic regions, untranslated regions (UTRs), and non-coding RNAs (ncRNAs). Research has found that a peptidogenomic strategy that integrates six-frame translation and mass spectrometry enabled genome-wide identification of these sORF-encoded peptides to further explore NCP function^[13]^. For instance, miPEPs are translated by ORFs in pri-miRNA sequences and enhance the transcription of their corresponding miRNAs to regulate target gene expression. In 84K poplar, functional characterization has shown that miPEP166i promotes adventitious root elongation^[14]^. Furthermore, uORFs in 5' UTRs typically inhibit downstream mORF translation^[15]^, as exemplified by MZF1-uPEP, which binds the zinc finger domain of YY1 to suppress aerobic glycolysis and neuroblastoma progression^[16]^. While peptides play critical regulatory roles in plants, their functions in woody species, remain largely unexplored. We hypothesize that the poplar genome encodes functional peptides, some of which are conserved across the *Populus* genus and perform similar regulatory functions, making their systematic identification in poplar a critically needed endeavor.

The 84K poplar (*P. alba × P. glandulosa*) has emerged as a key model system in forest genomics research owing to its rapid growth, environmental adaptability, and is readily transformable^[17]^. The 84K poplar genome is highly heterozygous (~2.1%)^[18]^, and its allelic resources are well-defined, with 29,295 allele pairs previously identified across its subgenomes^[14]^. Consequently, this system was selected to enable the identification and comparative analysis of peptides between its two haplotypes. Here, a comprehensive peptidogenomic analysis by integrating multi-tissue MS data from 84K poplar was performed to: (1) globally identify CPs and NCPs; (2) characterize their tissue- and allele-biased expression patterns; and (3) assess peptide conservation across six *Populus* species. This study provides a comprehensive repertoire of peptides, offering both molecular resources for functional peptide characterization and mechanistic insights into peptide-mediated regulation of forest growth and development.

## Materials and methods

### Sample preparation

Following a previously established protocol^[8]^, stem explants were obtained from 40-d-old *in vitro*-grown seedlings of the hybrid poplar clone 84K (*P. alba × P. glandulosa*) for vegetative propagation. Explants were cultured on half-strength Murashige and Skoog (1/2 MS) medium supplemented with 0.01 mg·L^−1^ naphthaleneacetic acid (NAA), and 0.05 mg·L^−1^ indole-3-butyric acid (IBA), under controlled conditions (temperature 23 ± 2 °C, photoperiod 16/8 h/light/dark, photosynthetic photon flux density (PPFD) 60 μmol·m^−2^·s^−1^), as previously described^[19]^. For sample collection, leaf tissues from 40-d-old seedlings were randomly selected for small RNA (sRNA) sequencing, while root, stem, and leaf tissues were collected for polypeptide extraction and identification. Each tissue type was sampled in three or four biological replicates. Specifically, three fully expanded leaves (excluding petioles) from the apical region of each seedling were harvested as leaf samples. Stem segments approximately 5–8 cm in length from the middle portion were used as stem samples, and all adventitious roots were collected as root samples. All fresh tissues were immediately snap-frozen in liquid nitrogen for 30 min and subsequently stored at −80 °C until further use.

### Peptide extraction

Approximately 1.0 g of quick-frozen poplar sample was weighed and transferred to a pre-chilled mortar. Liquid nitrogen was added, and the samples were ground to a fine powder. The powdered samples were then transferred to 1.5 mL microcentrifuge tubes, followed by the addition of lysis buffer containing 8 M urea. The mixture was sonicated on ice for 6 min (ultrasonic power: 20%; 2 s on/3 s off) to disrupt the cells. After sonication, the samples were centrifuged at 12,000 rpm for 15 min at 4 °C. The resulting supernatants were carefully collected and transferred to new microcentrifuge tubes. For polypeptide separation, the supernatants were loaded into a 10 kDa ultrafiltration tube and centrifuged at 11,000 rpm at 4 °C until fully filtered. The filtrate was then used for subsequent analysis. The separated peptides are stored at −80 °C for later use.

### Peptide desalination

For each biological replicate, peptide samples were individually desalted using C18 desalting columns. First, the columns were activated with 50% acetonitrile (ACN), followed by equilibration with washing buffer (0.1% formic acid [FA], 2% ACN), repeated four times. Next, the peptide solutions were loaded onto the equilibrated columns to allow peptide binding. The columns were then washed five times with washing buffer to remove impurities. Finally, bound peptides were eluted with elution buffer (0.1% FA, 60% ACN), and the eluates were collected into fresh microcentrifuge tubes. Each sample was subsequently vacuum-dried (or centrifuged to dryness) and stored at −80 °C until LC-MS/MS analysis.

### Peptide database construction

The genome assemblies of 84K poplar and six other *Populus* species, including *P. davidiana*, *P. deltoides*, *P. euphratica*^[20]^, *P. lasiocarpa*, *P. trichocarpa*, and *P. szechuanica*^[6]^were downloaded from databases in Supplementary Table S1. To construct a putative peptide database, six-frame translations were performed using the Sixpack tool from the EMBOSS suite (version 6.6.0)^[21]^. Each predicted peptide was defined as an ORF located between two stop codons, with a new peptide sequence initiated immediately after each stop codon. Following this definition, the resulting peptides were filtered to retain only those with lengths between 5 and 100 amino acids in the database. The genomic coordinates of all putative peptides were retained and stored in FASTA format for downstream analysis.

### Identification of CPs and NCPs

#### Peptide identification with PEAK studio

Raw mass spectrometry files were processed directly using PEAKS Studio software (version 8.5), with database searches performed against the custom potential peptide database of 84K poplar. The search parameters were configured with a precursor mass tolerance of 10 ppm and a fragment mass tolerance of 0.05 Da. To ensure high-confidence identifications, the results were stringently filtered as follows: Peptide-Spectrum Matches (PSMs) were filtered at a 1% False Discovery Rate (FDR); proteins were considered confidently identified only if they contained at least one unique peptide and passed the 1% FDR threshold at the protein level; and each identified peptide was required to be matched by at least two fragment ions in the MS/MS spectrum. These rigorous filtering criteria, applied to searches against the custom database, collectively ensured the reliability of the final peptide identifications.

#### Peptides identified in gene regions

To map the genomic origins of the candidate peptides, their coordinates (in BED format) were compared against the genome annotation (GFF format) using the intersect function from BEDTools (v2.30.0)^[22]^. To assign a unique genomic category to each peptide and eliminate redundant assignments, we applied a priority hierarchy: exon > intron > 5' UTR > 3' UTR > intergenic region. Gene bodies were defined by merging all coding sequences (CDS) for each gene, spanning from the start of the first CDS to the end of the last CDS. Based on this, the 1,000 bp region upstream of the gene body was defined as the 5' UTR, and the 500 bp region downstream was defined as the 3' UTR^[23]^. Intron coordinates were derived from exon intervals and cross-validated against the original GFF file. Based on these annotations, peptides mapping to exonic regions were classified as CPs, while all others were classified as NCPs.

#### Peptide characteristics calculation

The lengths of the peptides were recorded in FASTA format during the construction of the customized database. The molecular weight and isoelectric point of each peptide were calculated using the ProteinAnalysis.molecular_weight and ProteinAnalysis.isoelectric_point functions from the Biopython package^[24]^.

#### Peptide distribution at the genome level

Peptide distribution across chromosomes was visualized using the R package Rideogram^[25]^. Gene density was calculated using a sliding window of 0.1 Mb. Pearson correlation coefficients were computed to evaluate the relationship between chromosome length (with a 1 Mb window) and the number of CPs or NCPs. The genomic distance between adjacent peptides was calculated based on their coordinates within each chromosome, using a bin size of 1 Mb.

#### Tissue expression pattern of peptides

Tissue expression profile data were filtered using the criterion of mean ± 1.5 × standard deviation, and missing values in the filtered dataset were imputed using the mean value. Peptide expression trends that were co-expressed across all three tissues were analyzed using STEM, and a heatmap was generated for visualization. Genes encoding tissue-specific peptides were subjected to KEGG^[26,27]^ pathway enrichment analysis (based on *Arabidopsis thaliana*), and KEGG subcategory pathways were used for visual representation.

### Allele-level analysis of peptides

Results of allele pair identification in 84K poplar were obtained from previous research^[14]^. Based on allelic annotations, peptides were classified into two categories: those derived from the paternal parent, and those from the maternal parent. Reciprocal mapping was performed using BLAST^[28]^ (ncbi-blast-2.12.0) by aligning peptides of maternal origin to the paternal genome and vice versa. To eliminate redundancy, the two peptide sets were compared with each other using BLAST. Peptides were then categorized into shared peptides (G1), *alba*-specific peptides (G2), and *glandulosa*-specific peptides (G3). Genes encoding these peptides were subjected to KEGG pathway enrichment analysis using *Arabidopsis thaliana* as the reference. KEGG subcategory pathways were used for visual representation, and Fisher's method was applied to integrate *p*-values for enriched pathways.

### Conservation analysis of peptides in *Populus* species

Peptide databases for other *Populus* species were constructed using the same method applied to the customized peptide database of 84K poplar. To identify potential homologs of the 84K peptides in different species, we performed a BLAST search with a permissive E-value threshold of 1. This relaxed cutoff was chosen to maximize the inclusion of distantly related sequences, as standard E-value-based criteria are less effective for assessing short peptides. The results were further filtered using JCVI^[29]^ to retain only the top-scoring hit for each query peptide. Only mass spectrometry-validated, high-confidence peptides were included in the BLAST comparisons. To ensure high reliability in the conservation assessment, we applied stringent similarity criteria: peptide pairs were required to share > 90% sequence identity, with mismatches constituting < 10% of their length. These filters robustly identify highly conserved peptides while minimizing interference from interspecific annotation inconsistencies. Finally, Sequence LOGOs of six uORF-encoded peptides conserved across all six *Populus* species were generated using an online motif visualization tool^[30]^.

## Results

### Peptide identification in 84K poplar genome

**Figure 1:**
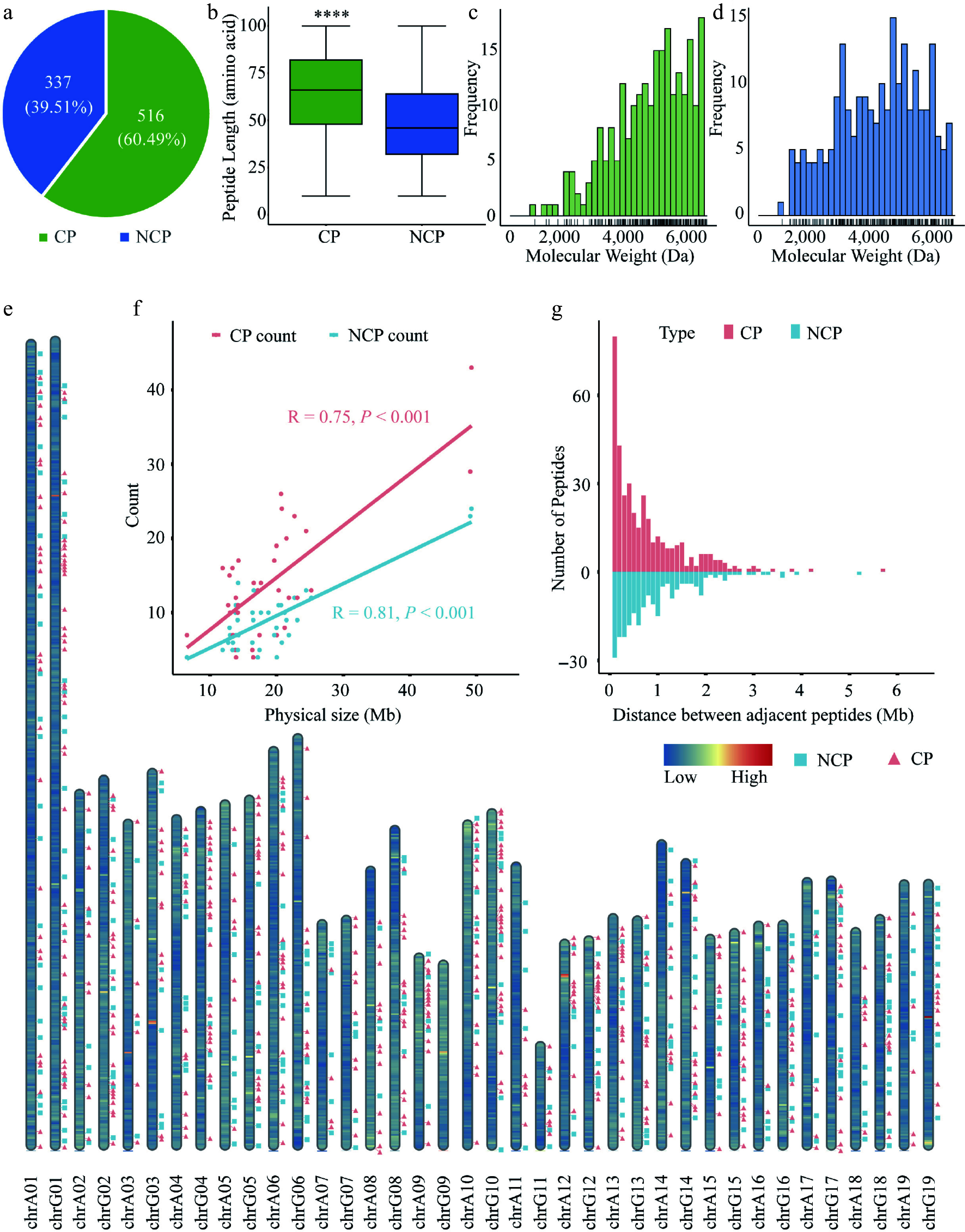
Overview and distribution of CPs and NCPs identified in poplar 84K. (a) Number of CPs and NCPs in poplar. (b) Length distribution of CPs and NCPs. Boxes represent the interquartile range (IQR, 25^th^ to 75^th^ percentile), whiskers extend to 1.5 × IQR. Wilcoxon's exact test was used for significance testing; *p* < 0.05. Molecular weight distribution of (c) CPs, and (d) NCPs, with rug plots indicating individual data points. (e) Genome-wide distribution of CPs (pink squares) and NCPs (green triangles) across chromosomes. Background color reflects gene density (blue: low; red: high). (f) Correlation between CP or NCP counts and chromosome length (Pearson's R: CPs = 0.75, *p* = 1.2e–07; NCPs = 0.81, *p* = 8.4e–10). (g) Distribution of distances between adjacent peptides.

Using LC-MS/MS combined with the customized poplar peptide database, we identified 853 unique sORF-derived peptides in root, stem, and leaf tissues of 84K poplar (Supplementary Table S2). Among them, 516 were CPs derived from exonic regions, and 337 were NCPs from non-coding regions, including introns, UTRs, and intergenic areas (Fig. 1a). The median length of CPs was 65 amino acids (aa), while that of NCPs was 45 aa (Fig. 1b). Furthermore, the average molecular weight of CPs was 7,084.22 Da, whereas that of NCPs was 5,797.67 Da (Fig. 1c, d).

Genomic distribution analysis revealed that both CPs and NCPs exhibit a clustered pattern across the 84K poplar genome (Fig. 1e). In addition, a strong positive correlation (Pearson's R > 0.7) was observed between peptide abundance and chromosome length, regardless of peptide origin (CPs or NCPs) (Fig. 1f). Analysis of inter-peptide distances showed a statistically significant difference in genomic spacing between CPs and NCPs (Wilcoxon rank-sum test, *p* = 0.0051). Across the genome, 92.3% of adjacent peptide pairs were located within 2 Mb of each other. Notably, CPs showed significantly higher density in proximal regions (< 1 Mb spacing) compared to NCPs (Fig. 1g).

### Characteristics of CPs and NCPs

**Figure 2:**
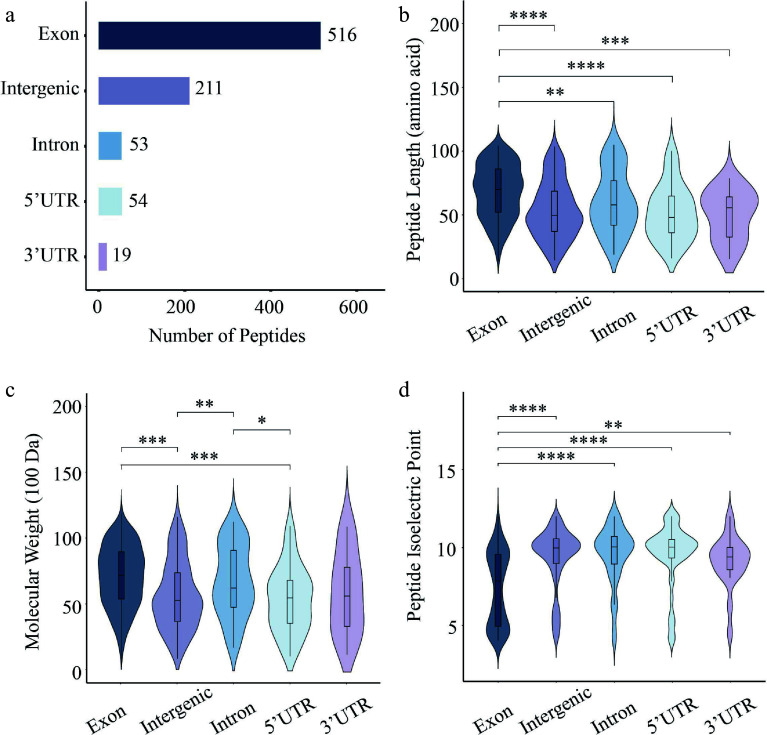
Characterization of 84K poplar peptides identified from exonic, UTR, intronic, and intergenic regions. (a) Number of peptides derived from different gene elements. Violin plots showing the distribution of (b) peptide length, (c) molecular weight, and (d) isoelectric point. Each plot combines a boxplot and a kernel density curve. Statistical significance was assessed using the Wilcoxon test (* *p* < 0.05, ** *p* < 0.01, *** *p* < 0.001, **** *p* < 0.0001).

Of the 853 identified peptides, 421 (49.3%) were derived from the forward strand, compared to 432 (50.7%) from the reverse strand (Supplementary Fig. S1). Regional annotation analysis showed that the majority of peptides (516, 60.5%) were CPs derived from exonic regions, while a substantial proportion (211, 24.7%) were NCPs from intergenic regions. Moreover, peptides derived from 3' untranslated regions (3' UTRs) were the least abundant, with only 19 identified (Fig. 2a). Comparative length analysis showed that CPs exhibited a significantly greater median length than NCPs from all other genomic regions (Fig. 2b**)**. Comparative analysis of molecular weight indicated that both CPs and intron-derived NCPs had significantly higher molecular weights than those from 5' UTRs and intergenic regions (Fig. 2c). The isoelectric point distribution of CPs was significantly different from that of NCPs derived from all four genomic regions (Fig. 2d).

### Analysis of tissue expression

**Figure 3:**
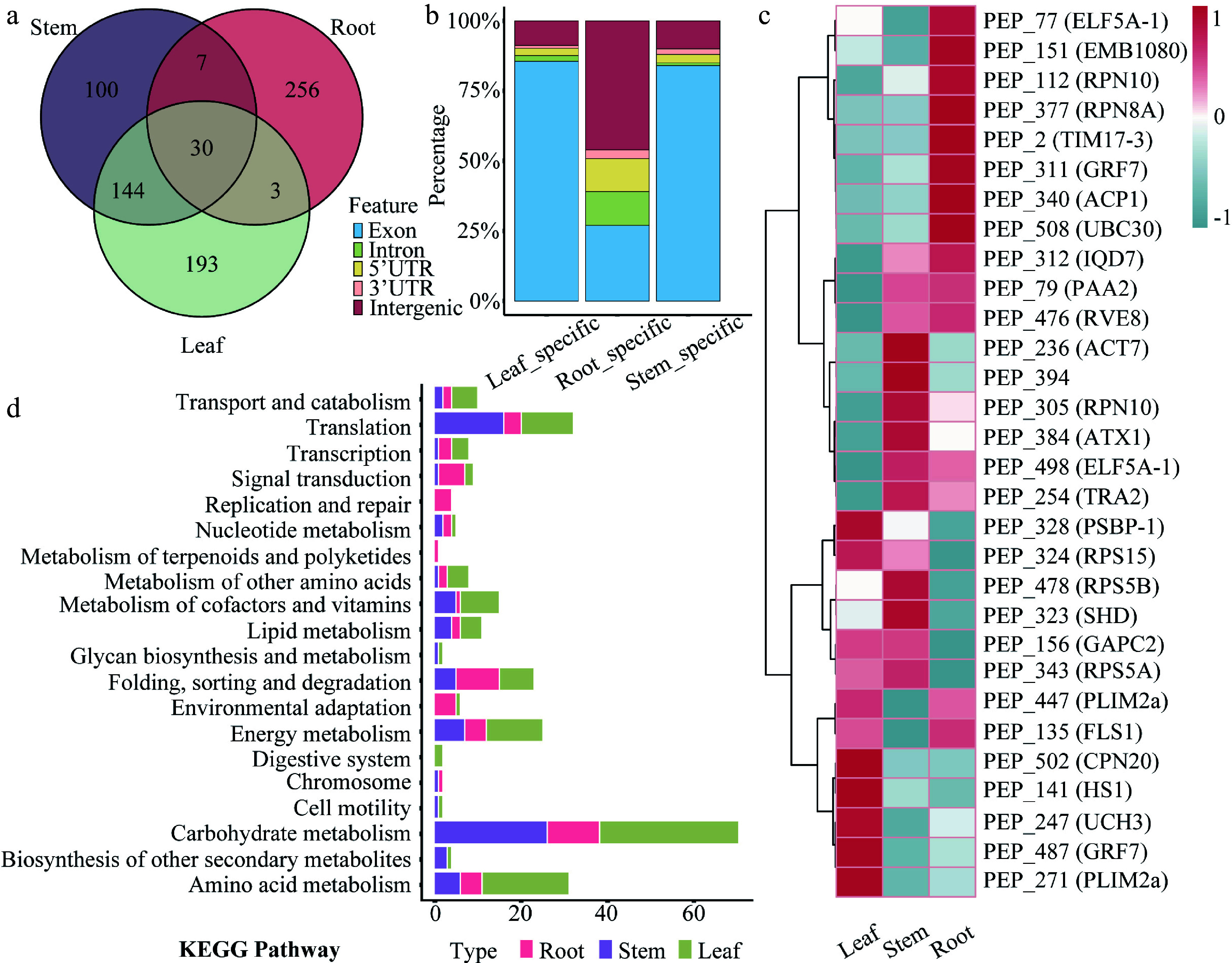
Tissue expression patterns of peptides in 84K poplar. (a) Venn diagram showing the number and overlap of tissue-specific peptides identified in roots, stems, and leaves. (b) Stacked bar chart displaying the percentage distribution of genomic origins of peptides with tissue-specific expression. (c) Heatmap presenting the expression profiles of 30 commonly expressed peptides across root, stem, and leaf tissues. Rows correspond to peptides, and columns to tissues. (d) KEGG pathway enrichment analysis of genes harboring tissue-specific peptide-associated ORFs. Bar lengths indicate the number of enriched genes, and bar colors represent corresponding tissues.

Tissue expression profiling identified 100 stem-specific peptides, 256 root-specific peptides, 193 leaf-specific peptides, and 30 CPs expressed ubiquitously across all three tissues (Fig. 3a and Supplementary Table S3). Among the tissue-specific peptides, CPs accounted for approximately 85% of both stem- and leaf-specific peptides. In contrast, only 25% of root-specific peptides were CPs, with a markedly higher proportion derived from intergenic regions. The combined proportion of stem- and leaf-specific peptides derived from 5' UTRs and introns was 4%–5%, whereas those from 3' UTRs comprised the smallest fraction (Fig. 3b).

Each of the four genes (*RPN10*, *PLIM2a*, *GRF7*, and *ELF5A-1*) contains two independent ORFs, with each ORF encoding a co-expressed CP. Expression trend analysis revealed tissue-biased patterns among these co-expressed peptides, despite their ubiquitous presence across tissues (Fig. 3c). Based on their expression dynamics, the peptides were grouped into nine distinct expression profiles. Most peptides clustered into Profile 4 and Profile 8, both exhibiting stem-preferential expression patterns (Supplementary Fig. S2). Furthermore, KEGG enrichment analysis of genes containing tissue-specific peptide-coding ORFs revealed enrichment in distinct biological pathways. Leaf-specific peptides were significantly enriched in photosynthesis and chloroplast-related metabolic pathways. Stem-specific peptides were predominantly enriched in pathways related to cell wall biosynthesis and secondary metabolism. Conversely, root-specific peptides showed predominant enrichment in nutrient uptake and rhizosphere microbial interaction pathways (Fig. 3d and Supplementary Table S4).

### Allele-biased expression and conservation of 84K-derived peptides in *Populus* species

**Figure 4:**
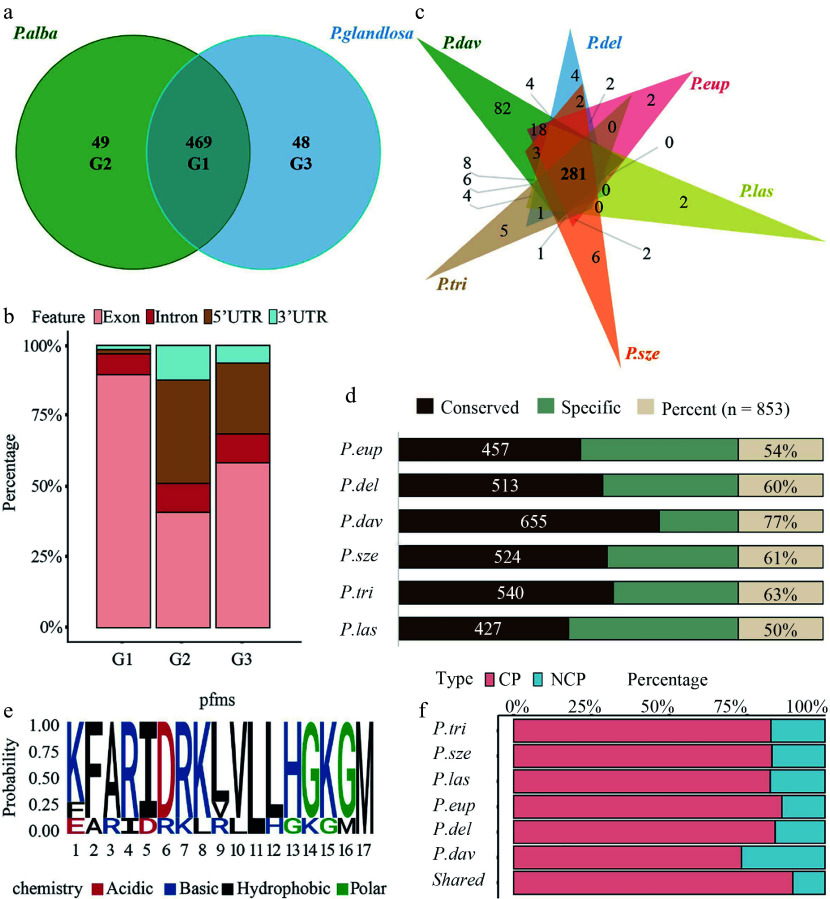
Allele-specific peptide mapping and cross-species conservation analysis. (a) Venn diagram illustrating the number and overlap of peptides identified through reciprocal mapping to the maternal (*P. alba*) and paternal *(P. glandulosa*) genomes. Group 1 (G1) represents peptides common to both parents, while Group 2 (G2) and Group 3 (G3) correspond to parent-specific peptides to *P. alba* and *P. glandulosa*, respectively. (b) Stacked bar chart showing the genomic origins of peptides across allelic categories, classified into exonic, 5′ UTR, intronic, and intergenic regions. (c) Venn diagram depicting the overlap of peptides identified in 84K poplar and six other *Populus* species (*P. euphratica*, *P.* *deltoides*, *P. davidiana*, *P. szechuanica*, *P. trichocarpa*, and *P. lasiocarpa*). (d) Bar chart showing the proportion of 84K poplar peptides conserved across the six *Populus* species. Conservation was assessed based on shared peptide sequences between 84K and each species. (e) Sequence LOGO of the PEP_629. Letters indicate amino acids, with height representing conservation levels; red indicates acidic, blue alkaline, black hydrophobic, and green polar amino acids. (f) CP and NCP percentage of identified 84K poplar peptides conserved in each *Populu*s species and in all *Populus* species.

Bidirectional alignment of the two haplotype genomes identified 469 shared peptides and 97 parent-specific peptides (49 *alba*-specific and 48 *glandulosa*-specific) (Fig. 4a and Supplementary Table S5). The 49 *alba*-specific peptides were encoded by ORFs located within 45 unique genes, while the 48 *glandulosa*-specific peptides originated from ORFs within 44 genes. In contrast, the 469 shared peptides were derived from ORFs spanning 435 genes (Supplementary Fig. S3a). Genomic origin analysis revealed that the shared peptides were predominantly CPs. However, a considerable proportion (20%–30%) of both *alba*-specific and *glandulosa*-specific peptides originated from 5' UTRs (Fig. 4b).

Functional enrichment analysis of genes containing ORFs for allele-biased peptides revealed distinct associations with biological processes. Genes harboring *alba*-specific peptide-encoding ORFs showed significant enrichment in amino acid metabolism and signal transduction pathways. *Glandulosa*-specific peptides showed predominant enrichment in carbohydrate metabolism and energy production pathways. In contrast, shared peptides were highly enriched in core biological processes such as translation and transcription (Supplementary Fig. S3b and Supplementary Table S6).

Given that approximately 83% of the peptides (excluding those from intergenic regions) were shared, their conservation across *Populus* species was further investigated. Homology analysis of 84K poplar peptides across six representative *Populus* species showed that 281 peptides (32.9%) shared highly homologous sequences (> 90%) across all six species (Fig. 4c and Supplementary Tables S7, S8). Moreover, *P. lasiocarpa* exhibited the lowest conservation (50%), whereas *P. davidiana* showed the highest (77%) (Fig. 4d). As shown in Fig. 4f, regardless of the *Populus* species, the proportion of conserved NCPs remained significantly lower than that of CPs. PEP_629 is an NCP translated from a uORF (Supplementary Table S9), exhibited conserved residues at positions 11 (leucine, L) and 17 (methionine, M) across seven *Populus* species, including 84K poplar, as revealed by sequence LOGO analysis (Fig. 4e). Both residues are hydrophobic amino acids. Including PEP_629, seven uORF-encoded peptides were conserved across *Populus* species (Supplementary Table S9). Sequence LOGO analysis of these peptides revealed relatively conserved amino acid patterns near their termination sites (Supplementary Fig. S4).

## Discussion

In recent years, our understanding of specific peptide functions has greatly expanded, and large-scale discoveries of NCPs have also been reported in diverse species, including maize^[31]^, *Arabidopsis*^[32]^, grape^[19]^, *Medicago truncatula*^[33]^, and rice^[34]^. Although certain studies have explored the functions of small peptides^[8]^, systematic investigations remain limited, and large-scale identification of these peptides in forest tree species is still scarce. In this study, we reported the first large-scale identification of CPs and NCPs in 84K poplar, revealing their tissue-specific expression profiles, allele-specific expression, and evolutionary conservation across *Populus* species.

### Species-specific characteristics of CPs and NCPs in 84K poplar

The present findings confirm the presence of peptides derived from non-coding genomic regions in 84K poplar, and conservation analyses suggest their likely existence in other *Populus* species (Fig. 4f). However, the total number of peptides identified is relatively small (< 1,000) (Fig. 1a), and the number of CPs exceeds that of NCPs, which is inconsistent with the findings of previous studies^[9,19]^. Furthermore, peptides identified in 84K poplar are, on average, 20–40 amino acids longer than those reported in maize, *Arabidopsis*, and grape, and they display a distinct molecular weight distribution^[9,19]^ (Fig. 1b). Another notable distinction is the strong positive correlation between CP abundance and chromosome length in 84K poplar (R = 0.75) (Fig. 1f), which was not observed in previous studies. These findings suggest that *Populus* species may utilize lineage-specific mechanisms to translate non-coding genomic regions. Supporting this hypothesis, 62.6% of the NCPs identified in 84K poplar originate from intergenic regions (Fig. 2a), which have traditionally been considered non-translatable, suggesting that these peptides may possess distinct and previously unrecognized biological functions.

### Contributions of CPs and NCPs to tissue specificity

In addition to those originating from intergenic regions, a significantly higher proportion of tissue-specific and allele-specific peptides were also derived from introns and 5' UTRs compared to other genomic features (Figs 3b and 4b). This observation suggests that these NCPs may play a regulatory role in mediating tissue-specific or allele-specific expression. In this study, we analyzed peptides derived from three primary tissues: roots, stems, and leaves. Leaves serve as the main photosynthetic organs, generating organic compounds that are essential for plant growth and development. They also facilitate gas exchange and transpiration^[35]^. Stems provide mechanical support and serve as conduits for the transport of water and nutrients throughout the plant^[36]^. Roots absorb water and mineral nutrients from the soil, supplying essential hydration and nutrition to the plant system. In addition, roots contribute to anchorage, nutrient storage, and phytohormone synthesis^[37]^. Enrichment analysis of genes containing tissue-specific peptide-coding ORFs indicated that their predicted functions correspond to the physiological roles of the respective tissues. In roots, where NCPs were highly abundant, pathway analysis revealed a dominant enrichment in nutrient uptake and rhizosphere interaction, underscoring the critical contribution of these peptides to root-specific biology. Owing to current technological constraints, our ability to detect a larger number of NCPs or to identify peptides co-expressed across multiple tissues was limited. Future studies could employ ribosome profiling to validate the presence and translational activity of NCPs in 84K poplar with greater resolution.

### Allele-specific and allele-biased expression and parental contributions

Allele-level expression has rarely been explored in previous studies involving other species. Given that 84K poplar is a hybrid, examining the parental origin of the identified peptides is essential. Our analysis revealed that nearly 21% of peptides were derived exclusively from only one parent, and clear allelic expression differences were observed in the genes harboring peptide-coding ORFs (Fig. 4a, b). Notably, the number of peptides originating from the paternal genome was approximately 27% higher than those from the maternal genome, suggesting a more substantial paternal contribution to the peptide landscape of 84K poplar (Fig. 4a). Such allelic expression differences may stem from variations in cis-regulatory elements, alternative RNA splicing, or promoter architecture^[38]^. A previous study has shown that DNA methylation affects allele-biased gene expression in 84K poplar^[14]^. This raises the possibility that DNA methylation may also influence allele-specific peptide expression, although further research is required for confirmation. Small peptides with allele-biased expression may be associated with heterosis or phenotypic divergence, and further studies are needed to elucidate how allelic variation contributes to their functional roles.

### Conservation of peptides and future directions

Most peptides were conserved not only between the two parental species of 84K poplar, but also across the six *Populus* species included in the analysis. These species, selected for conservation analysis, represent five major sections of the *Populus* genus, with the exception of *P. mexicana* (*sect. Abaso*). Conserved small peptides are often preserved by evolutionary selective pressures and may play critical roles in fundamental physiological or adaptive functions. These functional distinctions help explain the contrasting evolutionary patterns we observe: CPs engaged in essential, conserved processes exhibit stronger cross-species conservation, whereas NCPs enriched in environmentally responsive pathways such as nutrient uptake evolves more rapidly and shows lower conservation. This highlights the potential for future studies to investigate the core functions of these conserved peptides by integrating phenotypic data from different *Populus* species (Fig. 3d and Supplementary Fig. S3b).

Whether examining tissue-specific peptides, allele-specific peptides, or conserved peptides across *Populus* species, a consistent pattern emerges: NCPs show lower conservation and are more often associated with specialized biological functions (Fig. 4f). This observation is consistent with the properties of NCPs, such as miPEPs, previously reported in the literature^[39]^. Conversely, the higher conservation of CPs points to their fundamental roles in species evolution, while NCPs may contribute more to lineage-specific functional diversification. For instance, the *Zea*-specific peptide microRPG1 regulates grain dehydration, demonstrating that NCPs can constitute novel functional modules in plants. Our results also showed that highly conserved NCPs tend to originate from ORFs associated with core regulatory or stress-responsive pathways that are shared across *Populus* species. This suggests that these conserved NCPs might play roles in fundamental adaptive or physiological processes. For example, *RRC1* (*Reduced Red-light Responses in Cry1Cry2 Background 1*)^[40]^ contained a PEP612-encoding uORF; it was enriched in the spliceosome pathway and encodes splicing factors related to the alternative splicing function of light responsive genes. Similarly, *ABCG20* (*ATP Binding Cassette Subfamily G Member 20*)^[41]^ carried a PEP607-encoding uORF and was active in ABC-transporter and folate transport/metabolism pathways; its encoded peptide functions as an ABA-output protein that modulates *Populus* seed germination. And the gene *PAP1* (*Production of Anthocyanin Pigment 1*)^[42]^ harbored a PEP597-encoding uORF and participates in plant hormone signaling to control leaf color.

## Conclusions

This study presents the first large-scale identification of both conventional and non-conventional peptides in hybrid poplar 84K. A total of 853 sORF-encoded peptides were characterized, revealing distinct tissue-specific expression patterns and allele-biased origins. The lower conservation of NCPs across *Populus* species compared to CPs implies a potential role in lineage-specific adaptation. These findings provide a foundational peptide resource and a validated peptidogenomic framework for future functional studies in woody plants. Extending such profiling across diverse tree species will accelerate the discovery of conserved functional peptides for application in molecular forestry and breeding.

## SUPPLEMENTARY DATA

Supplementary data to this article can be found online.

## Data Availability

The datasets generated during and/or analyzed in the current study are available from the corresponding author on reasonable request.

## References

[1] (2004). Short-rotation woody crops and phytoremediation: opportunities for agroforestry?. Agroforestry Systems.

[2] (1996). Biology of *Populus* and its implications for management and conservation. Forest Science.

[3] (2006). The genome of black cottonwood, *Populus trichocarpa* (Torr. & Gray). Science.

[4] (2021). Genome-wide comparative analyses of GATA transcription factors among seven *Populus* genomes. Scientific Reports.

[5] (2022). Chromosome-scale assemblies of the male and female *Populus euphratica* genomes reveal the molecular basis of sex determination and sexual dimorphism. Communications Biology.

[6] (2024). The super-pangenome of *Populus* unveils genomic facets for its adaptation and diversification in widespread forest trees. Molecular Plant.

[7] (2000). Emerging model systems in plant biology: poplar (*Populus*) as a model forest tree. Journal of Plant Growth Regulation.

[8] (2024). A genome-wide identification of miPEPs in hybrid poplar reveals regulatory functions of miPEP166i in adventitious root elongation. Industrial Crops and Products.

[9] (2020). Large-scale discovery of non-conventional peptides in maize and *Arabidopsis* through an integrated peptidogenomic pipeline. Molecular Plant.

[10] (2019). Involvement of *Populus* CLEL peptides in root development. Tree Physiology.

[11] (2020). A xylem-produced peptide PtrCLE20 inhibits vascular cambium activity in *Populus*. Plant Biotechnology Journal.

[12] (2020). Peptide encoding *Populus CLV3/ESR-RELATED 47 (PttCLE47)* promotes cambial development and secondary xylem formation in hybrid aspen. New Phytologist.

[13] (2015). The plant peptidome: an expanding repertoire of structural features and biological functions. The Plant Cell.

[14] (2024). Allele-specific DNA methylation and gene expression during shoot organogenesis in tissue culture of hybrid poplar. Horticulture Research.

[15] (2014). Regulation of plant translation by upstream open reading frames. Plant Science.

[16] (2020). Therapeutic targeting of *YY1/MZF1* axis by MZF1-uPEP inhibits aerobic glycolysis and neuroblastoma progression. Theranostics.

[17] (2019). The genome of *Populus alba x Populus tremula* var. *glandulosa* clone 84K. DNA Research.

[18] (2024). High-quality genome assembly enables prediction of allele-specific gene expression in hybrid poplar. Plant Physiology.

[19] (2022). Large-scale discovery of non-conventional peptides in grape (*Vitis vinifera* L.) through peptidogenomics. Horticulture Research.

[20] (2020). Improved genome assembly provides new insights into genome evolution in a desert poplar (*Populus euphratica*). Molecular Ecology Resources.

[21] (2000). EMBOSS: the European molecular biology open software suite. Trends in Genetics.

[22] (2010). BEDTools: a flexible suite of utilities for comparing genomic features. Bioinformatics.

[23] (2013). Genome-wide analysis of DNA methylation in *Soybean*. Molecular Plant.

[24] (2009). Biopython: freely available Python tools for computational molecular biology and bioinformatics. Bioinformatics.

[25] (2020). *RIdeogram*: drawing SVG graphics to visualize and map genome-wide data on the idiograms. PeerJ Computer Science.

[26] (2023). KEGG for taxonomy-based analysis of pathways and genomes. Nucleic Acids Research.

[27] (2000). KEGG: Kyoto encyclopedia of genes and genomes. Nucleic Acids Research.

[28] (2009). BLAST+: architecture and applications. BMC Bioinformatics.

[29] (2024). JCVI: a versatile toolkit for comparative genomics analysis. iMeta.

[30] (2024). multiMotif: a generalized tool for scanning and visualization of diverse and distant multiple motifs. Journal of Genetics and Genomics.

[31] (2024). The *Zea mays* PeptideAtlas: a new maize community resource. Journal of Proteome Research.

[32] (2015). Primary transcripts of microRNAs encode regulatory peptides. Nature.

[33] (2017). Genome-wide identification of *Medicago* peptides involved in macronutrient responses and nodulation. Plant Physiology.

[34] (2024). Proteome-wide analysis reveals G protein-coupled receptor-like proteins in rice (*Oryza sativa*). Plant Signaling & Behavior.

[35] (2012). Leaf, stem and root tissue strategies across 758 Neotropical tree species. Functional Ecology.

[36] (2011). Plant stems: functional design and mechanics. Annual Review of Materials Research.

[37] (2018). Getting to the roots: a developmental genetic view of root anatomy and function from *Arabidopsis* to lycophytes. Frontiers in Plant Science.

[38] (2021). Perspectives on allele-specific expression. Annual Review of Biomedical Data Science.

[39] (2015). miRNA-encoded peptides (miPEPs): a new tool to analyze the roles of miRNAs in plant biology. RNA Biology.

[40] (2012). The RS domain of *Arabidopsis* splicing factor *RRC1* is required for phytochrome B signal transduction. The Plant Journal.

[41] (2019). *MtABCG20* is an ABA exporter influencing root morphology and seed germination of *Medicago truncatula*. The Plant Journal.

[42] (2005). Sucrose-specific induction of anthocyanin biosynthesis in *Arabidopsis* requires the *MYB75/PAP1* gene. Plant Physiology.

