# Supplementary material for: Identification and intra-genus conservation analysis of non-conventional peptides in hybrid poplar 84K
Source: For Res (Fayettev). 2026 Feb 28;6:e004. doi: 10.48130/forres-0026-0004 (PMC13187908; doi:10.48130/forres-0026-0004)
Supplement: Supplementary file 1 — Supplementary data to this article can be found online. [file forres-6-1-e004-Supplementary.zip › 10.48130_forres-0026-0004-Suppl-FigureS1 (1).pdf]

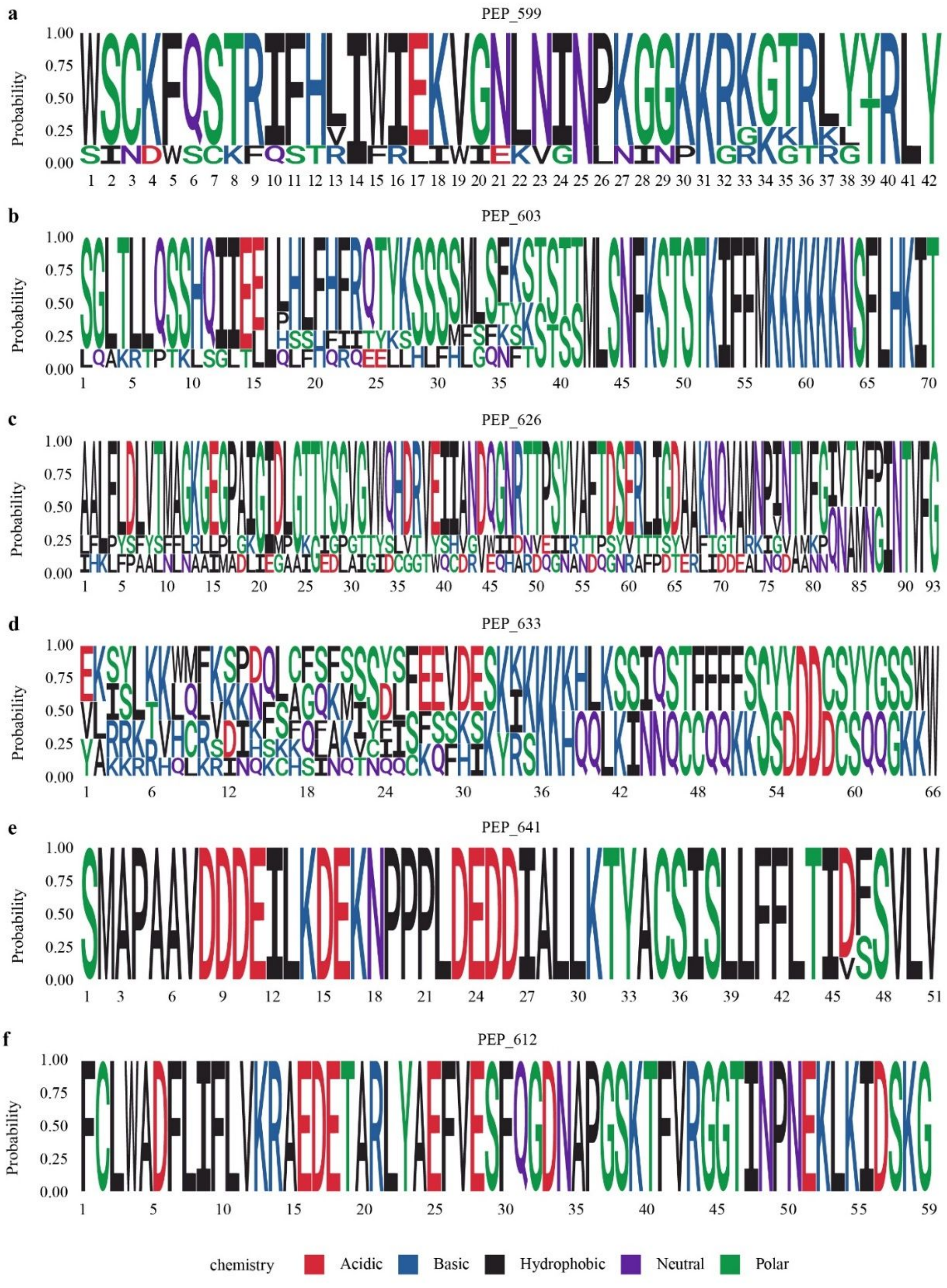

Fig. S4. LOGO analysis of conserved uORF peptides across six *Populus* species.

a-e Sequence LOGO analysis of 6 uORF encoded peptides which were conserved in 6 *Populus* species, letters represent amino acids, height represents conservation level, red represents acidic amino acids, blue represents alkaline amino acids, black represents hydrophobic amino acids, and green represents polar amino acids.
