# Supplementary material for: Identification and intra-genus conservation analysis of non-conventional peptides in hybrid poplar 84K
Source: For Res (Fayettev). 2026 Feb 28;6:e004. doi: 10.48130/forres-0026-0004 (PMC13187908; doi:10.48130/forres-0026-0004)
Supplement: Supplementary file 1 — Supplementary data to this article can be found online. [file forres-6-1-e004-Supplementary.zip › 10.48130_forres-0026-0004-Suppl-FigureS3.pdf]

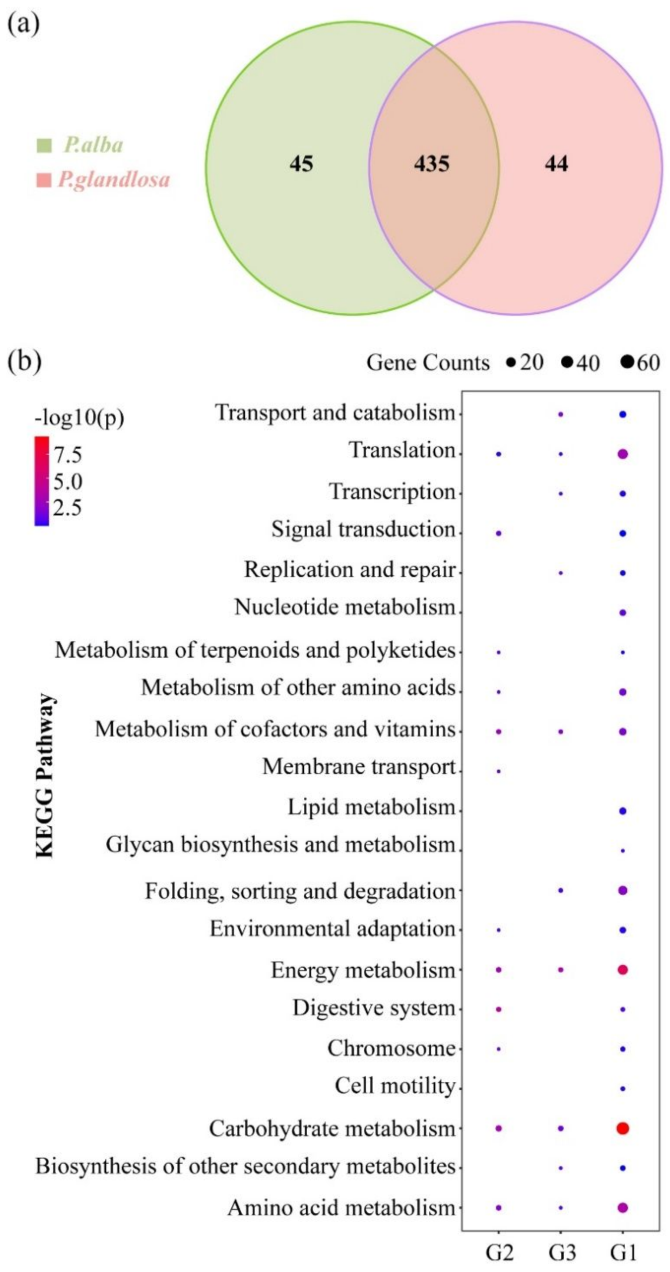

Fig. S3 Allelic origin and KEGG enrichment of ORF-hosting genes.

a Venn diagram showing the overlap of genes harboring peptide-associated ORFs identified in the maternal and paternal genomes. **b** KEGG pathway enrichment analysis of genes containing allele-specific or shared peptide-associated ORFs. Dot color represents the significance level of enrichment (-log10 p-value), and dot size indicates the number of enriched genes.
