# Supplementary figures and images for: Identification and intra-genus conservation analysis of non-conventional peptides in hybrid poplar 84K
Source: For Res (Fayettev). 2026 Feb 28;6:e004. doi: 10.48130/forres-0026-0004 (PMC13187908; doi:10.48130/forres-0026-0004)

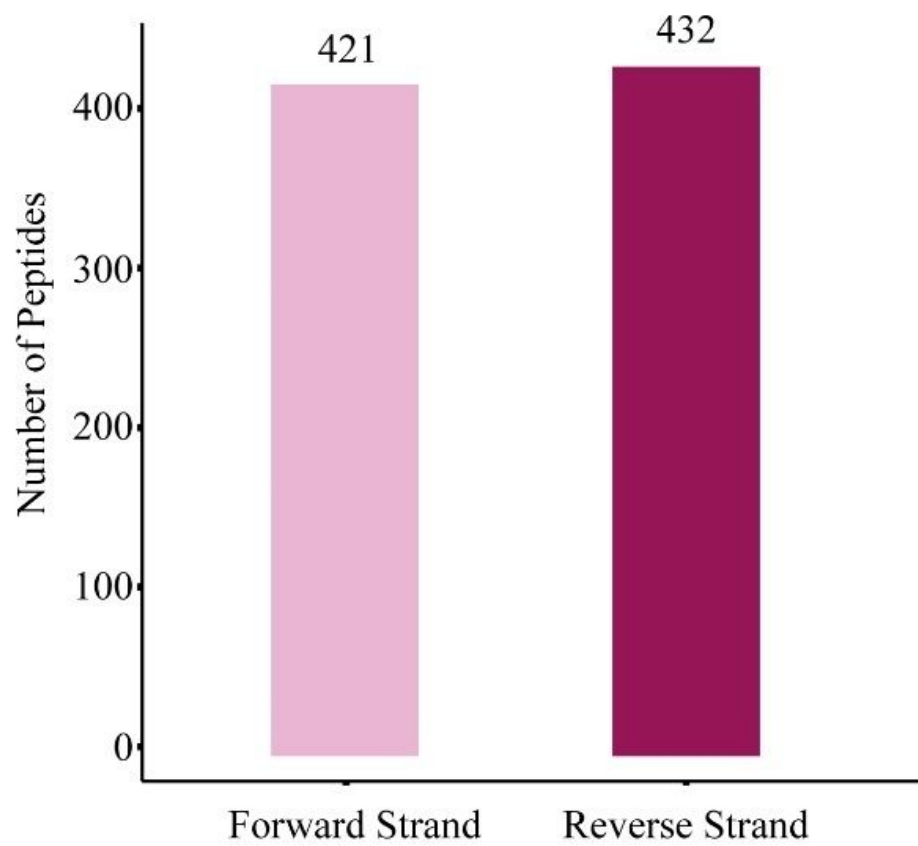

**Fig. S1 Number of peptides derived from forward or reverse strand.**

Supplement: Supplementary file 1 — Supplementary data to this article can be found online. [file forres-6-1-e004-Supplementary.zip › 10.48130_forres-0026-0004-Suppl-FigureS1.pdf]

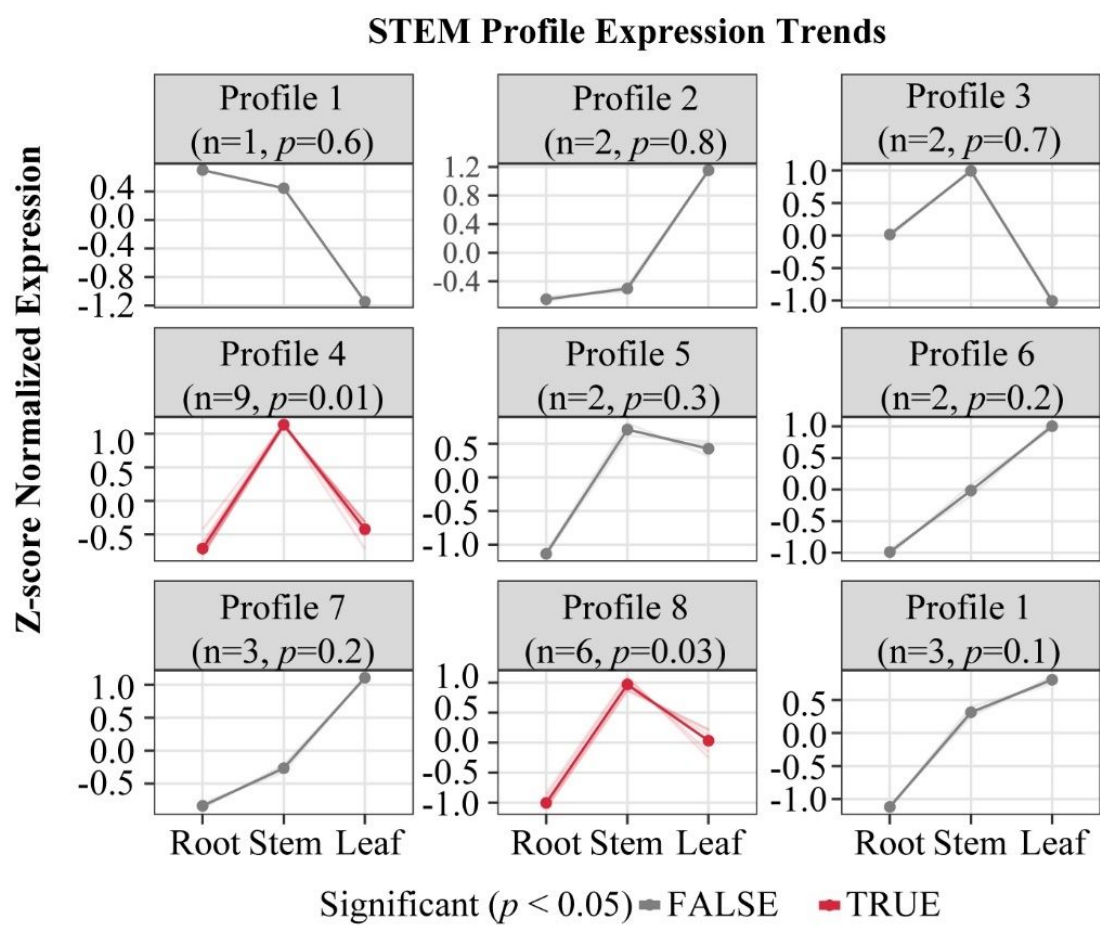

**Fig. S2 Clustered expression trends of shared peptides.**

Supplement: Supplementary file 1 — Supplementary data to this article can be found online. [file forres-6-1-e004-Supplementary.zip › 10.48130_forres-0026-0004-Suppl-FigureS2.pdf]
